# eHealth Literacy 2.0: Problems and Opportunities With an Evolving Concept

**DOI:** 10.2196/jmir.2035

**Published:** 2011-12-23

**Authors:** Cameron Norman

**Affiliations:** ^1^CENSE Research + DesignToronto, ONCanada

**Keywords:** eHealth literacy, measurement, consumer eHealth, social media

## Abstract

As the use of eHealth grows and diversifies globally, the concept of eHealth literacy – a foundational skill set that underpins the use of information and communication technologies (ICT) for health – becomes more important than ever to understand and advance. EHealth literacy draws our collective attention to the knowledge and complex skill set that is often taken for granted when people interact with technology to address information, focusing our attention on learning and usability issues from the clinical through to population health level. Just as the field of eHealth is dynamic and evolving, so too is the context where eHealth literacy is applied and understood. The original Lily Model of eHealth literacy and scale used to assess it were developed at a time when the first generation of web tools gained prominence before the rise of social media. The rapid shifts in the informational landscape created by Web 2.0 tools and environments suggests it might be time to revisit the concept of eHealth Literacy and consider what a second release might look like.

## Introduction

As the use of eHealth grows and diversifies globally, the concept of eHealth literacy – a foundational skill set that underpins the use of information and communication technologies (ICT) for health – becomes more important than ever to understand and advance [1]. eHealth literacy draws our collective attention to the knowledge and complex skill set that is often taken for granted when people interact with technology to address information, focusing our attention on learning and usability issues from the clinical through to population health level. Just as the field of eHealth is dynamic and evolving, so too is the context where eHealth literacy is applied and understood. The original Lily Model of eHealth literacy [1] and the scale used to assess it [2] were developed at a time when the first generation of web tools gained prominence before the rise of social media. The rapid shifts in the informational landscape created by Web 2.0 tools and environments suggests it might be time to revisit the concept of eHealth Literacy and consider what a second release might look like.

This issue of the *Journal of Medical Internet Research* (JMIR) provides examples of the diverse means in which the concept of eHealth Literacy has been applied, introducing challenges and presenting opportunities for understanding the evolution of the concept in the age of eHealth and mHealth. These challenges and opportunities will now be discussed in light of four papers published in this issue of JMIR [3-6].

## The eHealth Literacy Concept and eHEALS

The eHealth literacy concept, model and related measurement scale, the eHealth Literacy Scale (eHEALS) [1, 2], originated from work that Harvey Skinner and I were doing on ICT-facilitated health promotion with youth and youth workers in the late 1990’s and early 2000’s [7 -11]. At the time, those under the age of 25 were among the most prolific and creative users of ICT’s and thus, provided the ideal population to study the skill set required to access and fully engage with what became known as eHealth [12]. The concept of eHealth literacy was born of repeated observation in our research and health promotion practice that there was a noticeable gap between consumers’ absolute use of technology and the functional adoption of that technology into useful information finding and problem-solving. The eHEALS has been used in a variety of settings, with diverse population groups and has been translated into multiple languages [13-16]. The 8 or 10-item measure of eHealth Literacy continues to perform consistently across settings and populations.

## eHealth Literacy in This JMIR Issue

The theory and measurement of eHealth literacy is not without challenges and the papers in this issue highlight some of them, while presenting opportunities for further learning.

The work by Stellefson and colleagues [3] looked at the state of eHealth literacy research with young people, drawing on the original study and model of eHealth literacy by Norman and Skinner [1,2] and also the definition of health literacy posed by the U.S. Institute of Medicine [17]. The paper demonstrates how things can get conflated when looking at the literacy issues within an eHealth context. The original Lily Model – referred to by the authors in the introduction – posits that eHealth literacy is a form of meta-literacy, combining many different literacy skills beyond just health literacy or numeracy. To focus solely on just one or two aspects of literacy within the model when assessing how it manifests in practice is problematic when making claims about eHealth literacy as a whole given that the concept is intended to represent a set of integrated skills. eHealth literacy operates as part of a learning system, whereby the component parts operate as a whole and not in a means that is easily amenable to subdivision.

The study by Chan and Kaufman [6] illustrate this complexity while expanding the scope of how eHealth literacy is assessed in the practice of eHealth use. The authors look at the concept of eHealth literacy as it is expressed in the practice of information seeking and contribution to interactive discussions by looking at the task demands required to fully engage with eHealth. They propose a framework for characterizing the task demands associated with eHealth use and in doing so extend the eHealth literacy model in light of practice, offering to fill the gap between the theory and the measurement of the concept. While the eHEALS was cited in the paper, none of the selected studies used the eHEALS. Furthermore, the study’s inclusion criteria included papers that had “at least one aspect of eHealth literacy accounted for in the Norman and Skinner [1] definition used within this review”. In taking this approach, there is a risk that eHealth literacy is reduced to a set of interchangeable skills without attention to how they combine. Indeed, the argument posed when the model was created was that eHealth literacy was the combined features of the six forms of literacy or petals in the Lily Model, not a subset of them. 

Xie [5] took a different approach by looking at the eHEALS items along with measures of learning styles, preferences and general knowledge. The study focused on a population that has high needs for information, potentially greater isolation for informational resources, and a perceived lower familiarity with ICT’s. By looking at the skill set of eHealth literacy within a larger learning context, Xie reminds us that learning – no matter what the subject matter – is highly contextual, often social, and dependent upon learning styles and opportunities to connect with others.

Context is also an issue with the eHEALS itself, as the work by van der Vaart and colleagues [4] (perhaps unintentionally) introduce in their critique of the validity of the eHEALS. At the outset, the eHEALS was designed to be easy to use and administer in response to the expressed needs of health professionals who said they would not use a long instrument in practice. After three years of development, an 8 and 10-item version of the eHEALS was created to address research and health practitioner needs. The eHEALS was put through a rigorous testing process to explore the internal consistency reliability and validity of the instrument. In the initial studies that contributed to the development of the eHEALS, both reliability and validity scores were high, indicating that the measure was suitable to use. Since its initial testing and the publication of the eHEALS in JMIR, the eHEALS has been translated into multiple languages and employed with a diverse population stream from Chinese children [11] through to older adults [18]. While the results have been consistently positive, there are issues with the way that the concept of eHealth literacy has been measured.

The research by van der Vaart and colleagues [4] questions the validity of the eHEALS in light of a weak correlation between eHealth literacy and Internet use. The findings by van der Vaart and colleagues could be due to cultural differences, measurement inconsistencies across studies, or it could be due to something else related to the evolving nature of eHealth altogether. When the eHEALS was first developed, the correlation between the two was high, so what has changed and why might this latest research reveal something different? One of the principal reasons may be that the Internet has changed. When the eHEALS was first developed, the technology sector was in recovery from the dot-com bust and still seeking to develop itself. Social media hadn’t been realized, nor had widespread mobile Internet use taken off. Today, social media and the mobile web are among the most popular uses of the Internet among consumers [19]. While the eHEALS remains a strong tool for assessing competency with Web 1.0 related technologies, its fit with social media is unclear and the eHEALS feels incomplete. It is possible that these qualities are coming out with regards to current Internet use patterns, which are different and build on the foundational skills that the eHEALS measures. 

Such questions may be less about validity in a specific sense and more about the validity in a more generalized sense of Internet usage. Indeed, the positive coherence of the eHEALS in other studies and its psychometric robustness might suggest adding a social media interactive subscale rather than a change to the existing items, which would have significant consequences for the psychometric integrity of the instrument. Items could be developed that consider skills and tasks like:

- confidence in expressing oneself clearly in social interactions online
- ability to synthesize professional and non-professional advice
- comfort and skill in navigating through information obtained through a mobile device
- ability to use apomediaries to filter relevant and trustworthy information [20].

## Conclusion

The fundamental collection of skills that comprise eHealth Literacy have not likely changed, but the contexts in which they are expressed in the dynamic realm introduced by social media have. This presents an opportunity for research and practice to consider the ways in which eHealth literacy can be understood and fostered. The papers presented in this issue of JMIR provide important additions to the growing field of study in eHealth literacy and offer a glimpse as to where the concept may evolve to as more evidence unfolds.
